# Oleic Acid Induces Lung Injury in Mice through Activation of the ERK Pathway

**DOI:** 10.1155/2012/956509

**Published:** 2012-11-13

**Authors:** Cassiano Felippe Gonçalves-de-Albuquerque, Adriana Ribeiro Silva, Patrícia Burth, Isabel Matos Medeiros de Moraes, Flora Magno de Jesus Oliveira, Mauricio Younes-Ibrahim, Maria da Conceição Batista dos Santos, Heloísa D'Ávila, Patrícia Torres Bozza, Hugo Caire de Castro Faria Neto, Mauro Velho de Castro Faria

**Affiliations:** ^1^Laboratório de Imunofarmacologia, Instituto Oswaldo Cruz, FIOCRUZ, 21040-900 Rio de Janeiro, RJ, Brazil; ^2^Departamento de Biologia Celular e Molecular, Instituto de Biologia, Universidade Federal Fluminense, 24020-15 Niterói, RJ, Brazil; ^3^Departamento de Medicina Interna, Faculdade de Ciências Médicas, Universidade do Estado do Rio de Janeiro, 20550-900 Rio de Janeiro, RJ, Brazil; ^4^Departamento de Biologia Celular, Instituto de Ciências Biológicas, Universidade de Juiz de Fora, 36036-900 Juiz de Fora, MG, Brazil

## Abstract

Oleic acid (OA) can induce acute lung injury in experimental models. In the present work, we used intratracheal OA injection to show augmented oedema formation, cell migration and activation, lipid mediator, and cytokine productions in the bronchoalveolar fluids of Swiss Webster mice. We also demonstrated that OA-induced pulmonary injury is dependent on ERK1/2 activation, since U0126, an inhibitor of ERK1/2 phosphorylation, blocked neutrophil migration, oedema, and lipid body formation as well as IL-6, but not IL-1**β** production. Using a mice strain carrying a null mutation for the TLR4 receptor, we proved that increased inflammatory parameters after OA challenges were not due to the activation of the TLR4 receptor. With OA being a Na/K-ATPase inhibitor, we suggest the possible involvement of this enzyme as an OA target triggering lung inflammation.

## 1. Introduction

Adult respiratory distress syndrome (ARDS) description appeared in 1967, with 12 patients with refractory cyanosis to oxygenation therapy [1]. Recently, a draft definition proposed 3 mutually exclusive categories of ARDS based on degree of hypoxemia: mild (200 mm Hg < PaO2/FIO2 ≤ 300 mm Hg), moderate (100 mm Hg < PaO2/FIO2 ≤ 200 mm Hg), and severe (PaO2/FIO2 ≤ 100 mm Hg) [2] which was nominated as Berlin definition, replacing the American-European consensus [3]. The initial lesion characterizing the exudative phase of ARDS is an increase in alveolar permeability to plasma proteins, leading to an interstitial and alveolar oedema [4, 5]. In the acute phase, cytokines and lipids are released, leading to alveolar-capillary barrier loss with hyaline membrane formation [6, 7]. In fact, ARDS is a diffuse alveolar damage secondary to an intense lung inflammatory response to an infectious, noninfectious, or extra pulmonary insult [8, 9].

ARDS can be induced by several factors such as systemic endotoxin release, pneumonia, drug overdose, acid aspiration, fat embolism, and pancreatitis [10–13] and can occur in pathological processes including sepsis, major trauma, or severe leptospirosis [8, 14, 15]. 

Resolution of the pulmonary oedema and lung inflammation are important determinants of ARDS outcome. Removal of alveolar fluid depends on transport of salt and water across the alveolar epithelium through apical sodium channels (ENaC) followed by extrusion to the lung interstitium via the Na-K-ATPase of alveolar epithelial cells [16–18]. Oleic acid (OA) is an inhibitor of the Na/K-ATPase activity in bovine serum [19] and is also a Na/K-ATPase inhibitor in a rabbit lung model, increasing endothelial permeability [20]. OA has been reported to induce ARDS in experimental models [21] and lung injury correlates with elevated free fatty acid levels [22]. Furthermore, plasma and bronchoalveolar lavage fluid (BALF) from ARDS patients presented elevated OA levels [23, 24]. 

Lipopolysaccharide (LPS), an outer membrane component of Gram-negative bacteria, can cause serious multiple organ dysfunctions, particularly in the respiratory system [25]. LPS induces inflammation through the MAPKinase ERK1/2 activation. The ERK pathway participates in chemoattractant-induced neutrophil chemotaxis, in the respiratory burst [26] and in LPS-induced ARDS [27, 28]. The attenuation of ERK1/2 phosphorylation in neutrophils by luteolin has protected against the LPS-induced ARDS [29]. In alveolar macrophages, the combined inhibition of p38 and ERK1/2 induced a suppression of cytokine release [30]. In this respect, OA induces activation of the ERK1/2 pathway in a certain type of breast cancer cell [31]. 

We used a mouse model of OA-induced ARDS to study the involvement of ERK pathway in lung inflammation. We measured lung oedema, cell migration and activation, lipid mediator and cytokine production, lung morphological alterations, and the response to a respiratory functional test. 

## 2. Materials and Methods

### 2.1. Animals

We used male mice (30–35 g) of the following strains: Swiss Webster (SW), C57Bl/10 (from the Oswaldo Cruz Foundation Breeding Unit, Rio de Janeiro, RJ, Brazil) and C57Bl/10ScCr (kindly provided by the Federal Fluminense University Breeding Unit, RJ, Rio de Janeiro, Brazil). Animals were lodged at 22°C with a 12 h light/dark cycle and free access to food and water. Animal housing conditions and experimental procedures conformed to institutional regulations and were in accordance with the National Institute of Health guidelines on animal care. The institutional Animal Welfare Committee approved all procedures described here under license number 002-08. 

### 2.2. Preparation of Oleate Solutions

We used OA (from Sigma Chemicals) to prepare a 100 mM trisoleate solution. After weighting and water addition, trispowder (Trizma base-Sigma) was slowly added until the pH reached 10.0. This mixture was sonicated and, after complete oleate solubilization, the pH was carefully adjusted to 7.6 with diluted HCl. Working oleate solutions were prepared by appropriate dilutions of the 100 mM solution with sterile saline (PBS) pH 7.4. 

### 2.3. Intratracheal Oleate or LPS Administration

After isoflurane anesthesia, an incision above thyroid was made to expose the trachea. Trisoleate (1.25 *μ*mol in 0.05 mL), LPS (500 ng in 0.05 mL), or the same volume of saline (in controls) were instilled into the trachea of each mouse with an insulin syringe. After suturing the incision with a 3.0 silk thread, mice were returned to their cage and monitored until complete recovering from surgery. Inflammatory parameters were measured at different times after challenges. 

### 2.4. Total and Differential Cell Analysis in Bronchoalveolar Lavage Fluid (BALF)

After isolating the trachea by blunt dissection, 1.0 mL volume of PBS was instilled in each animal through a small caliber tube inserted into the airway. After gentle aspiration, 1 mL was recovered in each instillation/aspiration cycle. Total leukocyte counts were performed by microscopy in Neubauer chambers after diluting BALF samples in Türk solution (2% acetic acid). Differential leukocyte counts were done in cytocentrifuged smears stained with the May-Grunwald-Giemsa method. Total protein in BALF supernatants was determined using the Micron BCA Kit method (Pierce) according to the manufacturer's instructions.

### 2.5. Lipid Body Staining and Counting

While still moist, leukocytes on cytospin slides were fixed in 3.7% formaldehyde in Ca^2^+, Mg^2+^-free Hank's buffered salt solution ((HBSS), pH 7.4) and stained with 1.5% OsO_4_ [32]. Lipid bodies were enumerated by microscopy with oil-immersion objective lens in 50 consecutively scanned leukocytes. 

### 2.6. Cytokine/Chemokine Assays

Measurements of IL-6, CCL3/MIP-1*α*, TNF*α*, and IL-1*β* were done on cell-free BALF supernatants using ELISA kits, in accordance with the manufacturer's instructions (Duo Set, R&D Systems, Minneapolis, MN, USA).

### 2.7. PGE_2_ and LTB_4_ Assays

LTB_4_ and PGE_2_ in BALF supernatants were assayed by enzyme immunoassay (EIA) kits according to the manufacturer's instructions (Cayman Chemical, Ann Arbor, MI, USA). 

### 2.8. Morphologic Studies

24 h after the challenge with trisoleate or saline, animals were euthanized in a CO_2_ chamber and lungs were removed. For microscopy studies, lungs were fixed in 10% neutral buffered formalin, embedded in paraffin, sectioned at 4 *μ*m, and stained with hematoxylin and eosin.

### 2.9. Cell Culture Experiments

A549 lung epithelial cells were kindly provided by Dr. Cristina Plotkowski (from the Rio de Janeiro State University, Rio de Janeiro, RJ, Brazil). They were maintained in a complete DMEM/F12 (Hyclone) medium (containing 2% fetal bovine serum, 1% penicillin, and 100 UI/mL streptomycin). A day before the experiment, cells were treated with trypsin (0,025%), centrifuged at 4°C, 400 ×g for 10 min, resuspended in the complete medium, and incubated at 37°C in 5% CO_2_ in 24 well plates (300,000 cells per well). We tested two different OA concentrations (100 and 250 *μ*M in the final incubation volume). Cells were washed with PBS 30 min after the stimulus, lysed with lysis buffer (10 mM Tris pH 8.0, 150 mM NaCl, 1% Triton) containing protease inhibitors (Complete Protease Inhibitor Cocktail Tablets from Roche), and stored at −20°C.

### 2.10. Lung Tissue Experiments

Animals were anesthetized with ketamine and xylazine and then perfused with 20 mL of 20 mM ethylenediaminetetraacetic acid (EDTA) pH 7.4 through the right cardiac ventricle. Then, lung tissues cut into small pieces were homogenized at 4°C in a homogenizer using the lysis buffer containing protease inhibitors.

### 2.11. Evaluation of ERK1/2 Activation in Cultured Cells and Lung Tissues

Suspensions of cell and lung lysates in the electrophoresis sample buffer were heated at 100°C for 5 min and run in 10% polyacrylamide gels (PAGE-SDS). After transfer of gel proteins to nitrocellulose membranes under 15 V during 60 min (Biorad semidry system), membranes were incubated with a blocking solution followed by incubation with the monoclonal antibody antiphosphorylated ERK1/2 (Cell Signaling—1 : 1000 dilution) and then with the antimouse peroxidase conjugated antibody (Pierce, 1 : 10.000). The detection was performed with the “Super Signal Chemiluminescence” kit (Pierce), exposing the membrane to an autoradiograph film (Kodak MR Biomax). Membranes containing proteins were stripped, blocked again, and incubated with the monoclonal antibody antitotal ERK1/2 (Cell Signaling—1 : 1000) followed by treatment with antimouse antibody conjugated to peroxidase. After digitalized and analyzed by size and intensity by the Image Master 2D Elite 4.01 equipment, bands were compared to controls and normalized against total ERK1/2. Results' expression was in folds over controls.

### 2.12. Treatment with a MAP Kinase ERK1/2 Phosphorylation Inhibitor

In “*in vivo*” experiments, the ERK1/2 phosphorylation inhibitor U0126 (10 mg/kg), (1,4-diamino-2,3-dicyano-1,4-bis[2-aminophenylthio] butadiene) highly selective inhibitor of ERK 1 and ERK 2, was injected by the intraperitoneal route 30 min before OA administration. U0126 was previously dissolved in dimethylsulphoxide (DMSO) and diluted with PBS when used. 

### 2.13. Statistical Analysis

Results were expressed as mean ± SEM and analyzed by the Newman-Keuls-Student test. Differences were considered significant when *P* < 0.05.

## 3. Results

**Figure 1:**
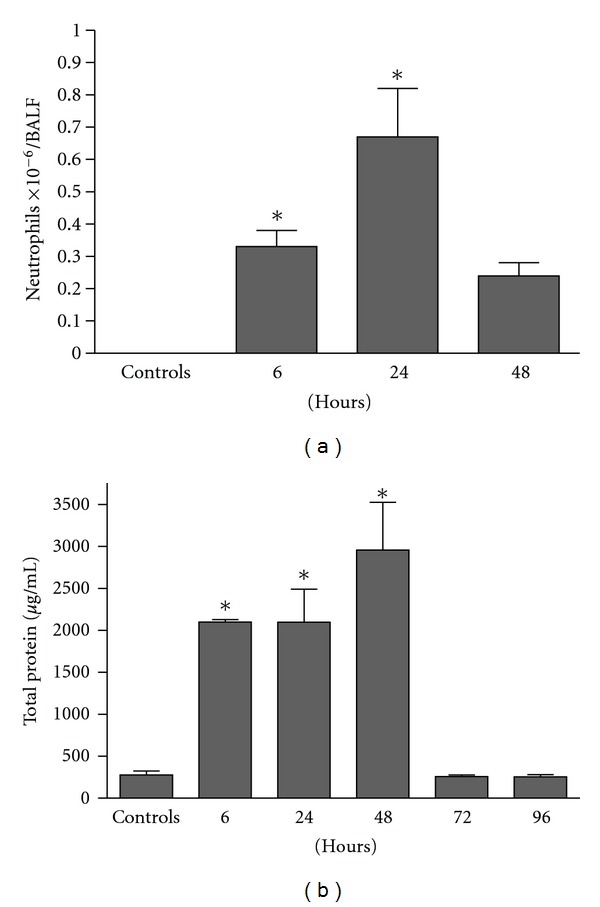
Intratracheal administration of OA induces lung inflammation in Swiss Webster mice. Kinetics of neutrophils (a) and protein accumulation (b) in BALF. Control groups received the same volume of PBS. Results are mean ± SEM of at least 7 animals. This experiment was repeated 3 times with similar results. *P < 0.05, compared to controls.

We used a mouse model of ARDS consequent to an intratracheal (IT) injection of oleic acid and showed that OA stimulation (1.25 *μ*mol per mouse, approximately 10 mg/kg of body weight) induced an intense neutrophil infiltration in SW mice. Cell migration was detected already at 6 h, peaked at 24 h, returning to basal levels at 48 h (Figure 1(a)). BALF total protein concentration, an indicator of oedema formation, increased in the first 6 h, remained high at 24 h and 48 h, but decreased thereafter (Figure 1(b)).

**Figure 2:**
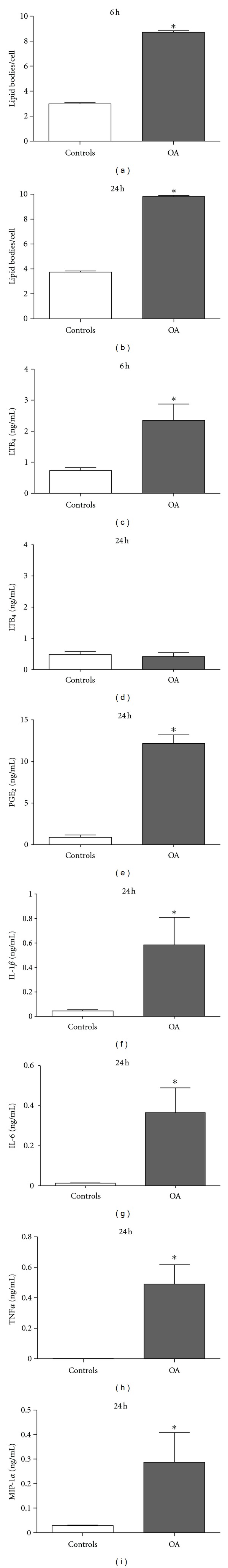
Intratracheal administration of OA induces leukocyte activation and production of inflammatory mediators in the lung of Swiss Webster mice. Lipid body counting was done at 6 h (a) and 24 h (b), while LTB_4_ measurements were done at 6 h (c) and 24 h (d). PGE_2_ (e), IL-1*β* (f), IL-6 (g), TNF*α* (h), and MIP-1*α* (i) were all measured 24 h after the challenge. Control groups received sterile saline. Each bar represents the mean ± SEM of at least 6 animals. *P < 0.05, compared to controls.

Lipid body formation, indicating cell activation, increased in BALF leukocytes 6 h (Figure 2(a)) and 24 h (Figure 2(b)) after OA administration. The lipid mediator LTB_4_ was significantly increased in BALF supernatant 6 h after the OA challenge (Figure 2(c)), decreasing to basal levels at 24 h (Figure 2(d)), while PGE_2_ reached its peak at 24 h (Figure 2(e)). In addition to inflammatory lipid mediators, we also measured cytokine concentrations (IL-1*β*, IL-6, TNF*α*, and MIP-1*α*) in BALF, showing that they were augmented 24 h after the OA stimulus (Figures 2(f), 2(g), 2(h), and 2(i), resp.).

**Figure 3:**
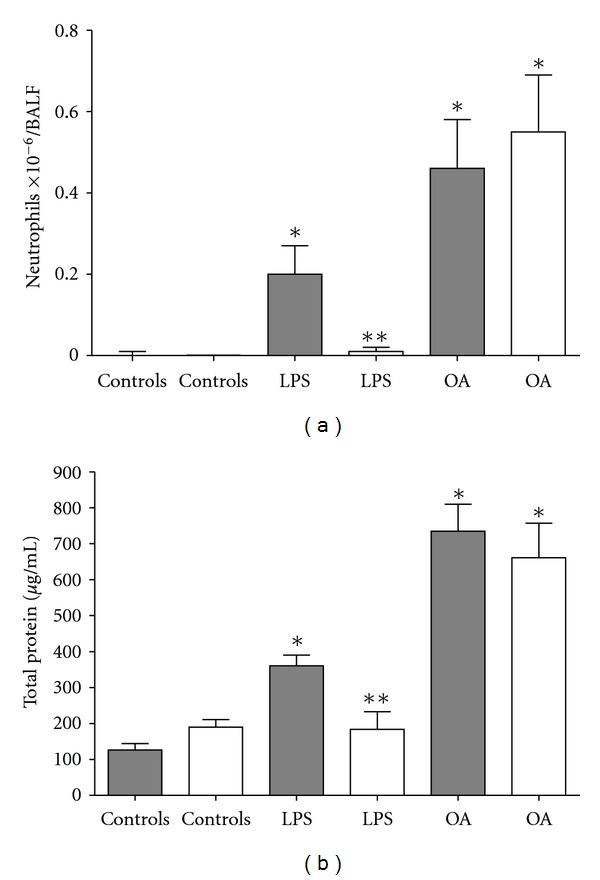
Intratracheal administration of OA failed to induce inflammation in TLR4 mutant C57B10/ScCr mice. Neutrophil (a) and protein (b) accumulation in BALF 24 h after stimulation of C57B10/ScCr (white bars) or C57B10 (dark bars) with OA and LPS. Control animals received PBS. Results are mean ± SEM from at least 11 animals. The experiment was repeated 3 times with similar results. *P < 0.05 compared to control group. **P < 0.05 C57B10/ScCr compared to C57B10 group for LPS.

We then compared OA and LPS challenges in C57BL10/ScCr and the corresponding wild-type C57BL10. C57BL10/ScCr mice possess a null mutation for TLR4 and are resistant to high LPS doses [33]. As expected, the wild-type strain showed a typical response to LPS, presenting cell migration and increased total protein in BALF, while the C57BL10/ScCr did not respond to LPS. On the other hand, OA not only induced cell migration but also augmented the total BALF protein in both animal types, as shown in Figures 3(a) and 3(b) then excluding a possible oleic acid contamination with LPS.

**Figure 4:**
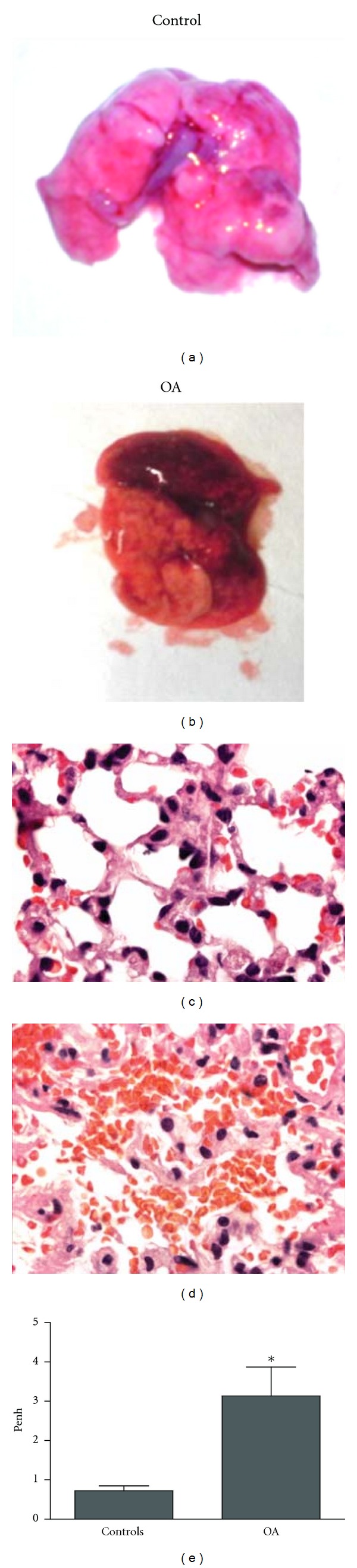
Representative macroscopic photo ((a) and (b)) and photomicrograph—1000x—((c) and (d)) of lungs from SW animals 24 h after OA challenge. Functional respiratory evaluation of animals 24 h after OA challenge using plethysmographic analysis (e). Controls received the same volume of PBS. Results of plethysmographic analysis are mean ± SEM from 7 animals. The experiment was repeated 3 times with similar results. *P < 0.05, compared to controls.

In Figures 4(a) and 4(b), macroscopic photos clearly show an intense hemorrhage in SW animals 24 hours after the OA injection, as compared to controls, while microscopic analyses (Figures 4(c) and 4(d)) of these lungs revealed an intense alveolar hemorrhage. Functional analysis by lung plethysmography using buxco revealed an altered pulmonary function 24 h after OA treatment (Figure 4(e)). 

**Figure 5:**
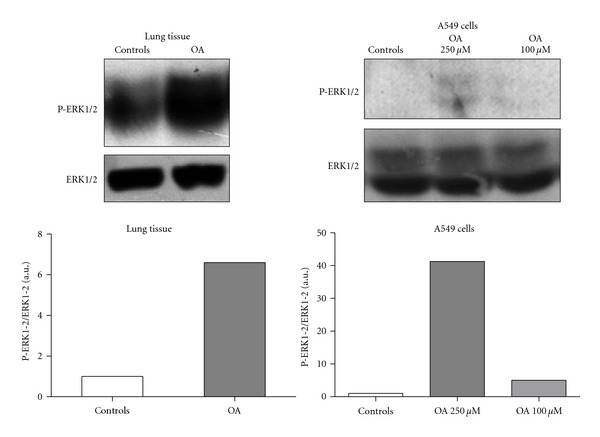
OA-induced ERK phosphorylation in the lung tissue and in A549 lung epithelial cells. Lungs were removed from SW animals 4** **h after the OA challenge. Cultured cell lysates were prepared after incubation for 30 min with 100 or 250 *μ*M OA. Graphics in this figure represent densitometric analyses of phosphorylated ERK1/2 and total ERK1/2 bands, as detailed in Methods.

We next evaluated the intracellular signaling mechanism involved in OA-induced inflammation. In our experiments, OA caused a substantial increase in MAPK ERK1/2 phosphorylation both *in vivo* and *in vitro*. Analysis of lung tissues 4 hours after IT injection of OA showed a marked increase in ERK1/2 phosphorylation. Cultured lung epithelial A549 cells also increased ERK1/2 phosphorylation after stimulation with OA (Figure 5). 

**Figure 6:**
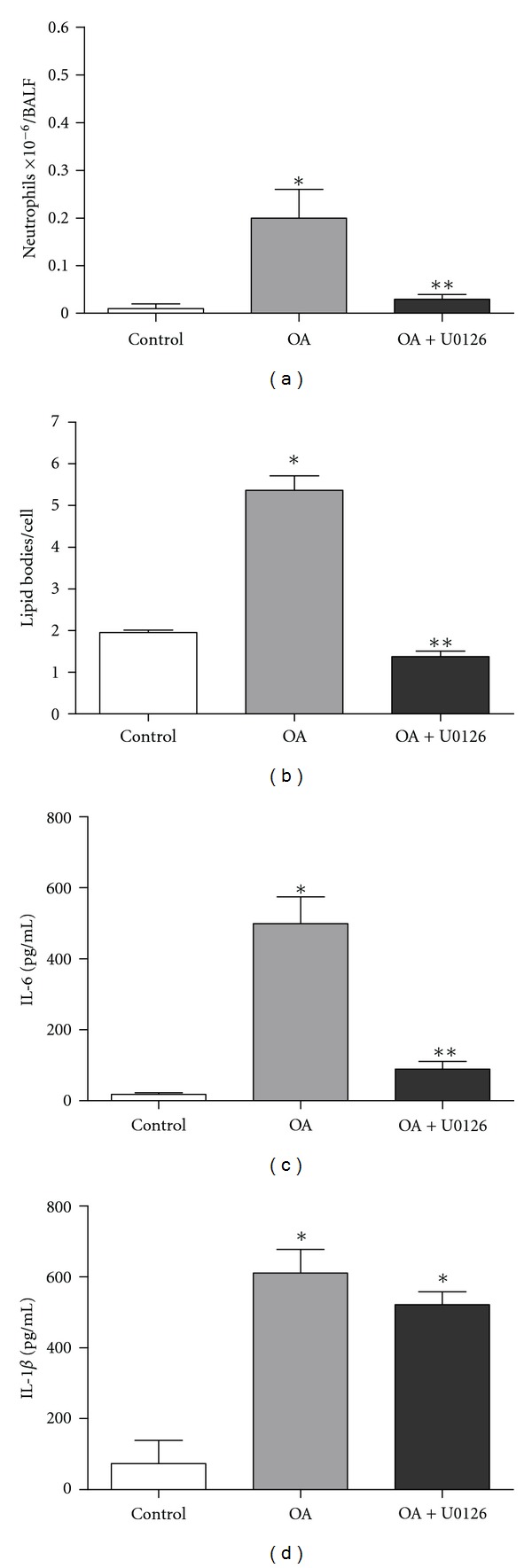
Blocking ERK1/2 activation by treatment with U0126 decreases OA-induced lung inflammation. Treatment protocols are described in Methods. Neutrophils (a) and lipid body formation (b). The following cytokines were also measured: IL-6 (c) and IL-1*β* (d). Controls received the same volume of saline. All measurements were performed 24** **h after OA challenge. Results are expressed as mean ± SEM from at least 6 animals. *P < 0.05 compared to control group; **P < 0.05 compared to OA stimulated group.

The role of ERK1/2 activation in our model was investigated by treating animals with the inhibitor of ERK1/2 phosphorylation U0126. Pretreating OA stimulated-SW mice with U0126 reduced significantly BALF inflammatory parameters as neutrophil migration (Figure 6(a)), lipid body formation (Figure 6(b)), and IL-6 synthesis (Figure 6(c)). IL-1*β* production (Figure 6(d)) was an exception, showing no significant reduction in U0126-treated animals.

## 4. Discussion 

In ARDS, neutrophil is the main cell migrating to lung [8, 34]. When activated, neutrophils release an arsenal of potent molecules contributing to increased tissue damage and inflammation [35]. In our experiments, neutrophil infiltration was already detected 6 h after OA injection, reaching a peak at 24 h and decaying to the basal level, thereafter indicating the resolution of the inflammatory process.

ARDS is described as an increase in endothelial permeability [21] and a loss of epithelial barrier function [13, 36], leading to pulmonary oedema. In ARDS, the transport capacity of the alveolar epithelium is greatly diminished and is correlated to a high mortality rate [37, 38]. Actions improving oedema clearance can offer important therapeutic options for patients with acute respiratory insufficiency. Na^+^ channels are pivotal in the control of Na^+^ clearance and recent data indicated that the increment of the Na/K-ATPase activity in type II cells was enough to increase the resolution of the alveolar fluid [16, 18, 39, 40]. Sodium channels contribute to alveolar fluid clearance under physiological conditions and the deregulation of the sodium channel activity might contribute to the pathogenesis of the pulmonary oedema [41]. OA is an Na/K-ATPase inhibitor [42–44] and also a sodium channel inhibitor [20]. Therefore, we suggest that, in our model, these mechanisms are involved in lung oedema formation. 

Cytokines such as TNF-*α* and interleukins (mainly IL-1*β* and IL-6) are important mediators in the development of ARDS [10], contributing to augmented vascular permeability and organ dysfunction [45]. IL-6 release seems to play a key role in ARDS [46] since IL-6 levels were found significantly higher in patients with subsequent ARDS than in patients who did not develop it [47], although its detailed mechanism of action remains unclear [48]. Accordingly, our results showed, in BALF supernatants of OA challenged animals, increased IL-6, IL-1*β*, and TNF-*α* production. The chemokine MIP-1*α*, a chemotactic factor for monocytes [49], was also increased, showing that intratracheal challenge with OA induces the main inflammatory mediators involved in clinical ARDS. The resulting inflammation could lead to the altered lung function seen by plethysmography.

Lipid bodies are cytoplasmic inclusions present in different cellular types [50]. In limited number, they are normal constituents of some cells, but they increase in number and size in those involved in inflammatory and immunologic processes [51, 52]. Lipid bodies generate the lipid mediators LTB_4_ and PGE_2_ [32]. In our model, oleic acid augmented the lipid body number as earlier as 6 hours after OA challenge, remaining high at 24 hours. Moreover, LTB_4_, a potent chemotactic agent for neutrophils [53], increased as early as 6 hours and could be the mediator involved in neutrophil migration into the lung in our model, returning to basal levels at 24 hours. PGE_2_ production was elevated 24 hours after OA challenge. In this regard, accumulating evidence suggests that the cyclooxygenase-2 (COX-2)/PGE_2_ pathway plays an important role in augmenting inflammatory immune response in sepsis-associated ARDS, since the inhibition of lung PGE_2_ production inhibits oedema, neutrophil infiltration, proinflammatory cytokine production, adhesion molecules expression, and restored lung morphology, increasing survival in polymicrobial sepsis [54]. Furthermore, this mediator inhibited phagocytosis and *in vitro* killing by alveolar macrophages, impaired lung recruitment of polymorphonuclear leukocytes, and also the clearance of *Streptococcus pneumoniae* [55].

In severe ARDS, hemorrhage can be present, as seen in patients with severe leptospirosis, leading to high mortality rates as a result of lung flooding and inflammation [56, 57]. Here we showed that hemorrhage was detected macroscopically and microscopically after OA challenge.

Toll-like receptors (TLR) participate in the detection of microorganisms. TLR4 is the ligand for LPS found in Gram-negative bacteria [58]. Using a mice strain carrying a null TLR4 mutation and comparing with its wild-type strain, we showed that OA was not acting through TLR4 activation. Therefore, oleic-acid-induced lung injury was not due to a LPS contamination of the oleic acid solution.

Extracellular signal-regulated protein kinase (ERK) was the first MAPK protein to be identified [59] and was subsequently shown to be involved in cell proliferation and activation [60]. In fact, OA induced ERK/MAPK phosphorylation in breast cancer cells [31] and also vascular smooth muscle cell proliferation and migration by a direct ERK-dependent mitogenic response [61, 62]. ERK is involved in regulating proinflammatory mediator production [30, 63]. We tested if OA could induce ERK1/2 phosphorylation in our model and this was effectively true not only in the lung tissue of mice, but also in cultured A549 epithelial lung cells. Specific inhibitor of RAS/ERK activity suppressed TGF-*β*1 production induced by oxidized low-density lipoprotein in human alveolar epithelial cells [63] and the U0126, a selective inhibitor of the mitogen-activated protein kinases MEK-1 and MEK-2, upregulated aquaporin 4 expression in alveolar type II cells in rats with oleic acid-induced lung injury [64]. We, therefore, investigated the impact of blocking ERK1/2 activation in OA-challenged mice, using the drug U0126 that inhibits the ERK phosphorylation step. Our results show that, in OA-challenged animals, ERK1/2 inhibition blocked neutrophil migration, oedema (data not shown), and lipid body formation as well as IL-6, but not IL-1*β* production. Since animals received OA directly into the lung, the direct epithelial cell damage due to the presence of this fatty acid could in part provoke lung injury. Nevertheless, the fact that U0126 inhibited OA-induced lung inflammation may argue against this possibility.

Considering that OA-induced inflammation is independent of TLR4 stimulation, a question rises about the primordial mechanism leading to ERK activation. Whereas OA is a Na/K-ATPase inhibitor, as already stated in the introductory section, and taking into account the signaling properties of this enzyme [65] promoting MAP kinase activation, including ERK [66], we suggest that Na/K-ATPase could be the initial OA target. 

IL-1*β* and IL-18 are produced as cytosolic precursors and they typically require secondary proteolytic cleavage induced by inflammasome for activation and secretion [67]. The inflammasome consists of several proteins, one of them, NLRP3, is involved in the recognition of bacterial RNA, ATP, uric acid, and low intracellular potassium concentration [68] which is a consequence of Na/K-ATPase inhibition by OA [69]. A recent communication showed that the leptospiral glycolipoprotein (GLP), which is also a specific Na/K-ATPase inhibitor [42, 43], activated the NLRP3 inflammasome by downregulating the Na/K-pump [70]. The inflammasome is an important step in IL-1*β* release, and, in our results, the IL-1*β* production was independent of ERK inhibition by U0126, pointing out, therefore, to a mechanism independent of ERK activation.

In conclusion, activation of the ERK signaling pathway independently of TLR4 stimulation is crucial in OA-induced lung injury. Furthermore, the participation of the Na/K-ATPase as a primary OA target in the mechanism of ERK activation is suggested.

## Abbreviations

OA: Oleic acid (18 : 1n-9)
ERK: Extracellular signal-regulated kinases
IL: Interleukin
TLR: Toll-like receptor
Na/K-ATPase: Sodium potassium ATPase pump
ARDS: Acute respiratory distress syndrome
LPS: Lipopolysaccharide
BALF: Bronchoalveolar lavage fluid
LTB_4_: Leukotriene B_4_
PGE_2_: Prostaglandin E_2_
TNF: Tumor necrosis factor
MIP-1*α*: Macrophage inflammatory protein 1 alpha.

## References

[1] Ashbaugh DG, Bigelow DB, Petty TL, Levine BE (1967). Acute respiratory distress in adults. *The Lancet*.

[2] Ranieri VM, Rubenfeld GD, Thompson BT (2012). Acute respiratory distress syndrome: the Berlin definition. *JAMA*.

[3] Bernard GR, Artigas A, Brigham KL (1994). The American-European Consensus Conference on ARDS: definitions, mechanisms, relevant outcomes, and clinical trial coordination. *American Journal of Respiratory and Critical Care Medicine*.

[4] Orfanos SE, Mavrommati I, Korovesi I, Roussos C (2004). Pulmonary endothelium in acute lung injury: from basic science to the critically ill. *Intensive Care Medicine*.

[5] Matthay MA, Zemans RL (2011). The acute respiratory distress syndrome: pathogenesis and treatment. *Annual Review of Pathology*.

[6] Nieuwenhuizen L, De Groot PG, Grutters JC, Biesma DH (2009). A review of pulmonary coagulopathy in acute lung injury, acute respiratory distress syndrome and pneumonia. *European Journal of Haematology*.

[7] Park WY, Goodman RB, Steinberg KP (2001). Cytokine balance in the lungs of patients with acute respiratory distress syndrome. *American Journal of Respiratory and Critical Care Medicine*.

[8] Ware LB, Matthay MA (2000). The acute respiratory distress syndrome. *The New England Journal of Medicine*.

[9] Pierrakos C, Karanikolas M, Scolletta S (2012). Acute respiratory distress syndrome: pathophysiology and therapeutic options. *Journal of Clinical Medicine and Research*.

[10] Artigas A, Bernard GR, Carlet J (1998). The American-European consensus conference on ARDS, part 2: ventilatory, pharmacologic, supportive therapy, study design strategies, and issues related to recovery and remodeling. *American Journal of Respiratory and Critical Care Medicine*.

[11] Brackenbury AM, Puligandla PS, McCaig LA (2001). Evaluation of exogenous surfactant in HCl-induced lung injury. *American Journal of Respiratory and Critical Care Medicine*.

[12] Gossling HR, Donohue TA (2003). The fat embolism syndrome. *Critical Care Medicine*.

[13] Matthay MA, Zimmerman GA (2005). Acute lung injury and the acute respiratory distress syndrome: four decades of inquiry into pathogenesis and rational management. *American Journal of Respiratory Cell and Molecular Biology*.

[14] Davidson KG, Bersten AD, Barr HA, Dowling KD, Nicholas TE, Doyle IR (2000). Lung function, permeability, and surfactant composition in oleic acid-induced acute lung injury in rats. *American Journal of Physiology*.

[15] Davidson KG, Bersten AD, Barr HA, Dowling KD, Nicholas TE, Doyle IR (2002). Endotoxin induces respiratory failure and increases surfactant turnover and respiration independent of alveolocapillary injury in rats. *American Journal of Respiratory and Critical Care Medicine*.

[16] Sznajder JI, Factor P, Ingbar DH (2002). Invited review: lung edema clearance: role of Na^+^-K^+^-ATPase. *Journal of Applied Physiology*.

[17] Vadász I, Morty RE, Olschewski A (2005). Thrombin impairs alveolar fluid clearance by promoting endocytosis of Na^+^,K^+^-ATPase. *American Journal of Respiratory Cell and Molecular Biology*.

[18] Matthay MA, Folkesson HG, Clerici C (2002). Lung epithelial fluid transport and the resolution of pulmonary edema. *Physiological Reviews*.

[19] Tal DM, Yanuck MD, Van Hall G, Karlish SJD (1989). Identification of Na^+^/K^+^-ATPase inhibitors in bovine plasma as fatty acids and hydrocarbons. *Biochimica et Biophysica Acta*.

[20] Vadász I, Morty RE, Kohstall MG (2005). Oleic acid inhibits alveolar fluid reabsorption: a role in acute respiratory distress syndrome?. *American Journal of Respiratory and Critical Care Medicine*.

[21] Schuster DP (1994). ARDS: clinical lessons from the oleic acid model of acute lung injury. *American Journal of Respiratory and Critical Care Medicine*.

[22] Rosen HR, Tuchler H (1992). Pulmonary injury in acute experimental pancreatitis correlates with elevated levels of free fatty acids in rats. *HPB Surgery*.

[23] Quinlan GJ, Lamb NJ, Evans TW, Gutteridge JMC (1996). Plasma fatty acid changes and increased lipid peroxidation in patients with adult respiratory distress syndrome. *Critical Care Medicine*.

[24] Bursten SL, Federighi DA, Parsons PE (1996). An increase in serum C18 unsaturated free fatty acids as a predictor of the development of acute respiratory distress syndrome. *Critical Care Medicine*.

[25] Mei SHJ, McCarter SD, Deng Y, Parker CH, Liles WC, Stewart DJ (2007). Prevention of LPS-induced acute lung injury in mice by mesenchymal stem cells overexpressing angiopoietin. *PLoS Medicine*.

[26] Kuroki M, O’Flaherty JT (1997). Differential effects of a mitogen-activated protein kinase kinase inhibitor on human neutrophil responses to chemotactic factors. *Biochemical and Biophysical Research Communications*.

[27] Nick JA, Young SK, Brown KK (2000). Role of p38 mitogen-activated protein kinase in a murine model of pulmonary inflammation. *Journal of Immunology*.

[28] Schuh K, Pahl A (2009). Inhibition of the MAP kinase ERK protects from lipopolysaccharide-induced lung injury. *Biochemical Pharmacology*.

[29] Lee JP, Li YC, Chen HY (2010). Protective effects of luteolin against lipopolysaccharide-induced acute lung injury involves inhibition of MEK/ERK and PI3K/Akt pathways in neutrophils. *Acta Pharmacologica Sinica*.

[30] Jarrar D, Kuebler JF, Rue LW (2002). Alveolar macrophage activation after trauma-hemorrhage and sepsis is dependent on NF-*κ*B and MAPK/ERK mechanisms. *American Journal of Physiology*.

[31] Soto-Guzman A, Robledo T, Lopez-Perez M, Salazar EP (2008). Oleic acid induces ERK1/2 activation and AP-1 DNA binding activity through a mechanism involving Src kinase and EGFR transactivation in breast cancer cells. *Molecular and Cellular Endocrinology*.

[32] Bozza PT, Payne JL, Morham SG, Langenbach R, Smithies O, Weller PF (1996). Leukocyte lipid body formation and eicosanoid generation: cyclooxygenase-independent inhibition by aspirin. *Proceedings of the National Academy of Sciences of the United States of America*.

[33] Poltorak A, He X, Smirnova I (1998). Defective LPS signaling in C3H/HeJ and C57BL/10ScCr mice: mutations in *Tlr4* gene. *Science*.

[34] Chen DL, Schuster DP (2004). Positron emission tomography with [^18^F]fluorodeoxyglucose to evaluate neutrophil kinetics during acute lung injury. *American Journal of Physiology*.

[35] Maniatis NA, Kotanidou A, Catravas JD, Orfanos SE (2008). Endothelial pathomechanisms in acute lung injury. *Vascular Pharmacology*.

[36] Matthay MA (1990). The adult respiratory distress syndrome: definition and prognosis. *Clinics in Chest Medicine*.

[37] Matthay MA, Wiener-Kronish JP (1990). Intact epithelial barrier function is critical for the resolution of alveolar edema in humans. *American Review of Respiratory Disease*.

[38] Ware LB, Matthay MA (2001). Alveolar fluid clearance is impaired in the majority of patients with acute lung injury and the acute respiratory distress syndrome. *American Journal of Respiratory and Critical Care Medicine*.

[39] Azzam ZS, Dumasius V, Saldias FJ, Adir Y, Sznajder JI, Factor P (2002). Na,K-ATPase overexpression improves alveolar fluid clearance in a rat model of elevated left atrial pressure. *Circulation*.

[40] Stern M, Ulrich K, Robinson C (2000). Pretreatment with cationic lipid-mediated transfer of the Na^+^K^+^-ATPase pump in a mouse model in vivo augments resolution of high permeability pulmonary oedema. *Gene Therapy*.

[41] Althaus M, Clauss WG, Fronius M (2011). Amiloride-sensitive sodium channels and pulmonary edema. *Pulmonary Medicine*.

[42] Burth P, Younes-Ibrahim M, Goncalez FHFS, Costa ER, Faria MVC (1997). Purification and characterization of a Na^+^,K^+^ ATPase inhibitor found in an endotoxin of Leptospira interrogans. *Infection and Immunity*.

[43] Younes-Ibrahim M, Buffin-Meyer B, Cheval L (1997). Na,K-ATPase: a molecular target for Leptospira interrogans endotoxin. *Brazilian Journal of Medical and Biological Research*.

[44] Swarts HGP, Schuurmans Stekhoven FMAH, De Pont JJHHM (1990). Binding of unsaturated fatty acids to Na^+^,K^+^-ATPase leading to inhibition and inactivation. *Biochimica et Biophysica Acta*.

[45] Sears BW, Stover MD, Callaci J (2009). Pathoanatomy and clinical correlates of the immunoinflammatory response following orthopaedic trauma. *Journal of the American Academy of Orthopaedic Surgeons*.

[46] Martín F, Santolaria F, Batista N (1999). Cytokine levels (IL-6 and IFN-*γ*), acute phase response and nutritional status as prognostic factors in lung cancer. *Cytokine*.

[47] Takala A, Jousela I, Takkunen O (2002). A prospective study of inflammation markers in patients at risk of indirect acute lung injury. *Shock*.

[48] Imai Y, Kuba K, Neely GG (2008). Identification of oxidative stress and Toll-like receptor 4 signaling as a key pathway of acute lung injury. *Cell*.

[49] Brueckmann M, Hoffmann U, Dvortsak E (2004). Drotrecogin alfa (activated) inhibits NF-kappa B activation and MIP-1-alpha release from isolated mononuclear cells of patients with severe sepsis. *Inflammation Research*.

[50] Dvorak AM, Morgan E, Schleimer RP, Ryeom SW, Lichtenstein LM, Weller PF (1992). Ultrastructural immunogold localization of prostaglandin endoperoxide synthase (cyclooxygenase) to non-membrane-bound cytoplasmic lipid bodies in human lung mast cells, alveolar macrophages, Type II pneumocytes, and neutrophils. *Journal of Histochemistry and Cytochemistry*.

[51] Dvorak AM, Dvorak HF, Peters SP (1983). Lipid bodies: cytoplasmic organelles important to arachidonate metabolism in macrophages and mast cells. *Journal of Immunology*.

[52] Bozza PT, Viola JPB (2010). Lipid droplets in inflammation and cancer. *Prostaglandins Leukotrienes and Essential Fatty Acids*.

[53] Samuelsson B, Dahlen SE, Lindgren JA (1987). Leukotrienes and lipoxins: structures, biosynthesis, and biological effects. *Science*.

[54] Ang S-F, Sio SWS, Moochhala SM, MacAry PA, Bhatia M (2011). Hydrogen sulfide upregulates cyclooxygenase-2 and prostaglandin E metabolite in sepsis-evoked acute lung injury via transient receptor potential vanilloid type 1 channel activation. *Journal of Immunology*.

[55] Medeiros AI, Serezani CH, Lee SP, Peters-Golden M (2009). Efferocytosis impairs pulmonary macrophage and lung antibacterial function via PGE_2_/EP2 signaling. *Journal of Experimental Medicine*.

[56] Marchiori E, Lourenço S, Setúbal S, Zanetti G, Gasparetto TD, Hochhegger B (2011). Clinical and imaging manifestations of hemorrhagic pulmonary leptospirosis: a state-of-the-art review. *Lung*.

[57] Marotto PCF, Ko AI, Murta-Nascimento C (2010). Early identification of leptospirosis-associated pulmonary hemorrhage syndrome by use of a validated prediction model. *Journal of Infection*.

[58] Medzhitov R, Preston-Hurlburt P, Janeway CA (1997). A human homologue of the *Drosophila* toll protein signals activation of adaptive immunity. *Nature*.

[59] Krishna M, Narang H (2008). The complexity of mitogen-activated protein kinases (MAPKs) made simple. *Cellular and Molecular Life Sciences*.

[60] Kim EK, Choi EJ (2010). Pathological roles of MAPK signaling pathways in human diseases. *Biochimica et Biophysica Acta*.

[61] Egan BM, Lu G, Greene EL (1999). Vascular effects of non-esterified fatty acids: implications for the cardiovascular risk factor cluster. *Prostaglandins Leukotrienes and Essential Fatty Acids*.

[62] Lu G, Morinelli TA, Meier KE, Rosenzweig SA, Egan BM (1996). Oleic acid-induced mitogenic signaling in vascular smooth muscle cells: a role for protein kinase C. *Circulation Research*.

[63] Guo L-L, Chen Y-J, Wang T (2012). Ox-LDL-induced TGF-*β*1 production in human alveolar epithelial cells: involvement of the Ras/ERK/PLTP pathway. *Journal of Cellular Physiology*.

[64] Chen CL, Li TP, Zhu LH (2009). Effect of MAPK signal transduction pathway inhibitor U0126 on aquaporin 4 expression in alveolar type II cells in rats with oleic acid-induced acute lung injury. *Nan Fang Yi Ke Da Xue Xue Bao*.

[65] Xie Z, Cai T (2003). Na^+^-K^+^-ATPase-mediated signal transduction: from protein interaction to cellular function. *Molecular Interventions*.

[66] Edwards A, Pallone TL (2007). Ouabain modulation of cellular calcium stores and signaling. *American Journal of Physiology*.

[67] Martinon F, Burns K, Tschopp J (2002). The Inflammasome: a molecular platform triggering activation of inflammatory caspases and processing of proIL-*β*. *Molecular Cell*.

[68] Akira S, Uematsu S, Takeuchi O (2006). Pathogen recognition and innate immunity. *Cell*.

[69] Morth JP, Pedersen BP, Buch-Pedersen MJ (2011). A structural overview of the plasma membrane Na^+^,K^+^-ATPase and H^+^-ATPase ion pumps. *Nature Reviews Molecular Cell Biology*.

[70] Lacroix-Lamandé S, D'Andon MF, Michel E (2012). Downregulation of the Na/K-ATPase pump by leptospiral glycolipoprotein activates the NLRP3 inflammasome. *Journal of Immunology*.

